# Performance of interferon-γ release assays in the diagnosis of confirmed active tuberculosis in immunocompetent children: a new systematic review and meta-analysis

**DOI:** 10.1186/s12879-016-1461-y

**Published:** 2016-03-18

**Authors:** Patrizia Laurenti, Matteo Raponi, Chiara de Waure, Marta Marino, Walter Ricciardi, Gianfranco Damiani

**Affiliations:** Institute of Public Health - Section of Hygiene, Università Cattolica del Sacro Cuore, Largo Francesco Vito, 1-00168 Rome, Italy

**Keywords:** Active tuberculosis, Children, Meta-analysis, Diagnosis, Tuberculin skin test, IGRAs, QuantiFERON-TB Gold In-Tube, T-SPOT.TB

## Abstract

**Background:**

Tuberculosis (TB) is a global public health problem, causing morbidity and mortality in adults and children. The most reliable diagnostic tools currently available are the in vivo Tuberculin Skin Test (TST) and the *ex vivo* Interferon-γ release assays (IGRAs). Several clinical, radiological, and bacteriological features make the detection of active (overt disease) TB in children difficult. Although recently developed immunological assays such as QuantiFERON-TB Gold In-Tube (QFT-IT) and T-SPOT®.TB are commonly used to identify active TB in adults, different evidence is required for diagnosis in children. The purpose of this study was to reassess the sensitivity and specificity of IGRAs in detecting microbiologically confirmed active TB in immunocompetent children.

**Methods:**

A systematic review and meta-analysis of studies reporting on the diagnostic accuracy of tests for TB in immunocompetent children aged 0–18 years, with confirmation by positive *M. tuberculosis* cultures, were undertaken. Electronic databases were searched up to September 2015 and study quality assessment was performed using QUADAS-2.

**Results:**

Fifteen studies were included in our meta-analysis. Results showed that there were no significant differences in sensitivity between TST (88.2 %, 95 % confidence interval [CI] 79.4–94.2 %), QFT-IT (89.6 %, 95 % CI 79.7–95.7 %) and T SPOT (88.5 %, 95 % CI 80.4–94.1 %). However, both QFT-IT (95.4 %, 95 % CI 93.8–96.6 %) and T-SPOT (96.8 %, 95 % CI 94.2–98.5 %) have significantly higher specificity than TST (86.3 %, 95 % CI 83.9–88.6 %).

**Conclusions:**

QFT-IT and T-SPOT have higher specificity than TST for detecting active TB cases in immunocompetent children.

## Background

Tuberculosis (TB) is one of the most important global public health problems and one of the major causes of adult and childhood morbidity and mortality worldwide. In 2012, there were an estimated 530,000 TB cases (bacteriologically confirmed or clinically diagnosed) among children <15 years of age, approximately 6 % of the total number of 8.6 million cases. Among HIV-negative children, there were 74,000 TB-related deaths, approximately 8 % of the total number of 940,000 TB-related deaths among HIV-negative people [1].

In 2011, the trend in the pediatric TB notification rate showed a slight decline during the previous ten years from a peak of 5.7 in 2001. However, a number of countries, such as Bulgaria, Finland and Italy, have seen increasing trends during the same period [2]. Indeed, across Europe during the period 2000–2009, a decline or stabilization of trends was reported in high-incidence countries while low-incidence countries tended to report an increased incidence in pediatric TB.

In 2009, only 19.2 % of all childhood TB cases in Europe were confirmed by culture, a clear indication that TB diagnosis in children remains a major public health challenge [3]. Several clinical, radiological and bacteriological features (such as pauci-bacillary nature, atypical clinical signs, and a lower probability of bacteriological confirmation) make the detection of active TB in children difficult, often leading to the neglect of TB within pediatric populations [4].

As a result, the diagnosis of active disease in children often relies on a combination of contact history, clinical symptoms, and radiological findings, together with a consideration of the results of a Tuberculin Skin Test (TST) [5, 6].

The most reliable diagnostic tools currently available for identifying TB infection are the in vivo TST and the *ex vivo* interferon-γ (IFN-γ) release assays (IGRAs). For almost 100 years, the TST was the main test of choice for identifying TB infection. This test measures an individual’s response to a solution of *Mycobacterium tuberculosis* antigens and can produce false-positive and false-negative responses due to immunologic immaturity or cross-reactivity with mycobacteria not in the *M. tuberculosis* complex, vaccination with Bacille Calmette-Guérin (BCG), and other undetermined causes [7, 8]. Within the past decades, however, two new immunological assays have been developed: the QuantiFERON-TB Gold (QFT-G; Qiagen), QuantiFERON-TB Gold In-Tube (QFT-IT; Qiagen), and the T-SPOT®.TB assay (Oxford Immunotec). QFT-G and QFT-IT measure the concentration of IFN-γ produced in whole blood by enzyme-linked immunosorbent assay (ELISA) [8, 9]. T-SPOT measures the number of individual *Mycobacterium*-specific T cells secreting IFN-γ by the enzyme-linked immunosorbent spot (ELISPOT) assay [10, 11].

In adults, a higher specificity of IGRAs compared with TST has been reported. The sensitivity for active TB ranges from 70 to 90 % and is lower in high TB incidence settings [12–15]. Thus, IGRAs are now included by the CDC in the recommended diagnostic algorithm for detection of TB in adults [16]. However, caution is recommended regarding their use in children [17].

A growing number of studies have compared TST and IGRAs for the detection of *M. tuberculosis* infection, a condition that may or may not progress to clinical disease and active (overt disease) TB in children. Studies have measured sensitivity in populations with active TB and in populations exposed to TB cases [18, 19]. Six meta-analyses [6, 20–24] have previously assessed IGRAs’ sensitivity and specificity in children and reported largely different pooled estimates. These differences are due to the characteristics of the study populations and different inclusion/exclusion criteria (such as immunologic status, level of income, and concurrent infections). Two of these previous meta-analyses focused on either bacteriologically confirmed or clinically diagnosed TB cases [6, 22], one included contacts with TB cases in addition to the previous two categories [20], another also included cases of latent TB [21] and one [23], although providing a sub-analysis on microbiologically confirmed cases, included studies for which it was not possible to clearly identify methods used to confirm cases. In the last meta-analyses, which provided a sub-analysis including only microbiologically confirmed cases, the study population also included immunocompromised children [24]. Because of this heterogeneity, pooled estimates of sensitivity and specificity of IGRAs and TST have varied considerably. Through the use of different inclusion/exclusion criteria compared with previous studies, the aim of our study was to reassess the sensitivity and the specificity of IGRAs, QFT-IT, and T-Spot TB versus TST in the detection of bacteriologically confirmed active TB in immunocompetent children aged 0–18 years.

## Methods

### Literature retrieval

An extensive search of the scientific literature was carried out by querying electronic databases of PubMed, EMBASE and Cochrane Library to identify articles published in English or Italian between January 1^st^ 2003 and September 30^th^ 2015. The following terms were used as keywords: “tuberculosis”, “tuberculosis infection”, or “tuberculosis disease”; “pediatrics” or “child*”; “Tuberculin Test”; “Interferon-gamma Release Tests”, “QuantiFERON”, “ELISpot”, “QFT- IT”, “QFT-2G”, “IFN”, “T-cell assays”, “T-SPOT.TB test”, “ESAT-6”, “CFP10”, or “RD1 antigens”; “Sensitivity”; and “Specificity”. Further retrieval of grey literature was conducted through consulting Google Scholar and websites of the World Health Organization (http://www.who.int/en/), Centre for Disease Control and Prevention (http://www.cdc.gov/) and the National Institute for Health and Clinical Excellence (https://www.nice.org.uk/) for relevant unpublished studies and national and international guidelines. We integrated the electronic searches with manual searches, checking the reference lists of relevant articles to identify further studies.

### Selection criteria

Potential studies were selected through consideration of the title and abstract by two researchers. Disagreements were solved by a senior researcher. Full texts of eligible articles were read by two researchers to decide upon final inclusion.

The following inclusion criteria were used: only studies performed on healthy children from 0 to 18 years were considered eligible, and articles which included only adults or immunosuppressed children (such as HIV-positive patients) were excluded; we included only those studies focused on the sensitivity and specificity of IGRAs or TST in detecting confirmed active TB cases (considered as a child with active TB disease, confirmed by positive *M. Tuberculosis* cultures); we included only those studies including sensitivity and specificity, or where it was possible to calculate them; we included only articles that reported original data (reviews, case reports and editorials were excluded); and we included only those studies with ≥5 study subjects.

### Quality assessment

Two independent researchers evaluated the validity of the selected studies using the Revised Quality Assessment of Diagnostic Accuracy Studies (QUADAS-2) tool [25]. This tool assesses the risk for bias and concerns regarding applicability in four domains: patient selection, index test, reference standard, and study flow and timing. The risk of bias was evaluated through the identification of specific questions and the development of guidance on items evaluation according to QUADAS-2 recommendations. The reviewers recorded and compared the answers given to each question.

Both reviewers analysed all articles in terms of the study population, index test, reference standard, setting, diagnostic pathway, target condition, and flow diagram. For each article, researchers independently recorded a score of “low risk of bias/low concerns regarding applicability,” “high risk of bias/high concerns,” or “unclear” for each question. All domains with at least one negative response scored “high risk of bias” (if the negative response regarded the risk for bias) or “high concerns regarding applicability” (if the negative response regarded the applicability), while domains with no negative responses but at least one unsure response scored “unclear”. Domains with no negative and no unsure responses scored “low risk of bias/low concerns”. All disagreements were resolved by consensus.

### Data abstraction and data analysis

Data were extracted using a standardized form including the following information: authors, year of publication, journal, country, country TB burden, study design, age of the patients, sample size, TB diagnostic tests, and TST cut-off. For each study, children representing true positives (TP), true negatives (TN), false positives (FP), and false negatives (FN) were defined by identifying microbiological culture as the reference test. With respect to TST, patients were classified as positive or negative according to the TST cut-off chosen by both the authors of each paper and to all three TST cut-offs defined by the American Academy of Paediatrics (AAP) (>5 mm, >10 mm, >15 mm). The three cut offs suggested by the AAP were applied to all patients of each study because it was not possible to classify patients in risk groups as defined by the AAP itself.

Two authors independently extracted data from the papers and corroborated their findings. Pooled sensitivity and specificity of TST, QFT-IT and T-SPOT and a 95 % confidence interval (CI) were calculated using the Der Simonian and Laird random effects model. Furthermore summary receiving operating characteristic (sROC) curves with Area Under the Curve (AUC) were obtained on the basis of the Littenberg and Moses model. Meta-Disc, version 1.4 (Hospital Ramony Cajal, Madrid, Spain) [26] was used to perform the analysis. A value of 0.5 was added to all cells in studies where any cell was 0. Heterogeneity was assessed using the I^2^ statistic. Pooled likelihood positive and negative ratios (LR+ and LR-) were obtained to assess the informative power of the three tests.

## Results

### Literature search

**Fig. 1:**
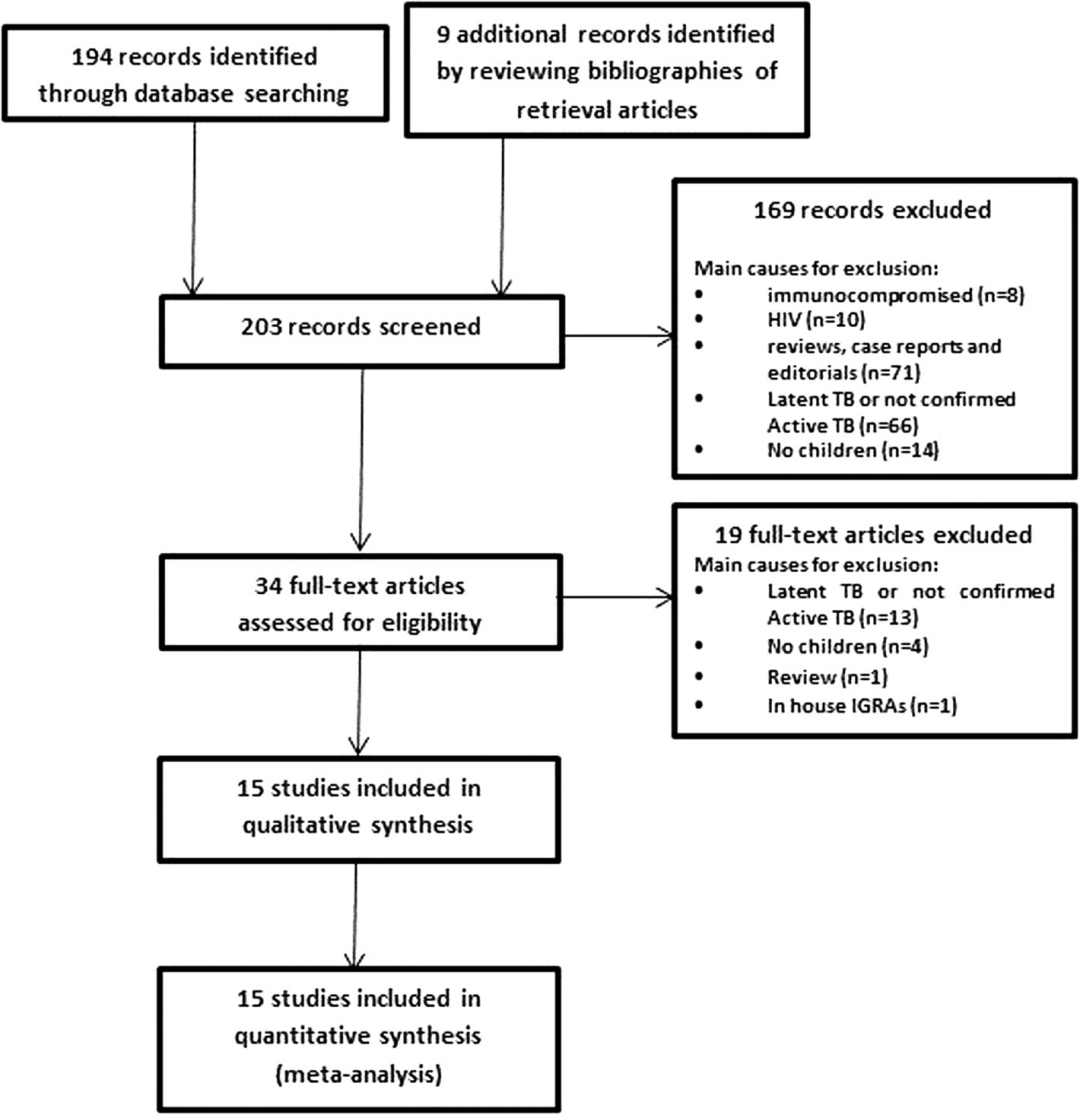
Flow chart of included studies

A total of 194 articles were obtained through database searching. Among them, 169 articles were excluded after abstract reading and a further 19 excluded after review of the full text. Furthermore, after reviewing references of retrieval articles, an additional nine studies were included. A total of 15 studies [27–41] were included in the systematic review (Fig. 1).

The included studies were undertaken in 11 countries, of which four (Lithuania, China, India and Uganda) [31, 33, 37, 39, 41] had a high TB burden. Among studies considered in the analysis, six assessed both the sensitivity and specificity of IGRAs and TST [27, 30, 31, 33, 36, 39]. The TST cut-off was set at 10 mm in nine studies, 5 mm in four studies, 15 mm in one study, and not defined in one. Regarding the IGRAs, five assessed both QFT-IT and T-SPOT.TB, seven only QFT-IT, and three only T-SPOT.TB. Characteristics of all included studies are given in Table 1.

**Table 1:** Characteristics of included studies

Author, Year	Journal	Country	Study design	Age (years, m = months)	Sample size - TB cases - Controls	TB diagnostic test	Sensitivity	Specificity	TST cut-off (mm)	TB burden
Detjen et al. (2007) [27]	Clin Infect Dis.	Germany	cohort study	4 m-15	28 22	TST	1^a^	1^a^	>10	Low
						QFT-IT	0.93^a^	1^a^		
						T-SPOT.TB	0.93^a^	1^a^		
Domínguez et al. (2008) [28]	Clin Vaccine Immunol.	Spain	cohort study	≤18	9	TST	1^a^	–	≥5	Low
						QFT-IT	0.67^a^	–		
						T-SPOT.TB	0.86^a^	–		
Kampmann et al. (2009) [29]	Eur Respir J.	United Kingdom	cohort study	3 m-16	25	TST	0.88	–	≥10	Low
						QFT-IT	0.80	–		
						T-SPOT.TB	0.58	–		
Lighter et al. (2009) [30]	Int J Tuberc Lung Dis.	USA	cohort study	≤17	7 21	TST	0.86^a^	0.86^a^	≥10	Low
						QFT-IT	0.86^a^	1^a^		
Hansted et al. (2009) [31]	BMC Pulm Med.	Lithuania	cohort study	10–17	23 52	TST	1	0.35^a^	≥10	High
						T-SPOT.TB	1	0.90^a^		
Bamford et al. (2010) [32]	Arch Dis Child.	United Kingdom	cross-sectional study	7.2 m-16	49	TST	0.82	–	>15	Low
						T-SPOT.TB	0.67	–		
						QFT-IT	0.78	–		
Sun Lin et al. (2010) [33]	Chinese Medical Journal	China	case–control study	≤18	18 51	TST	0.61	0.71	≥10	High
						T-SPOT.TB	0.83	0.94		
Tsolia et al. (2010) [34]	Pediatr Infect Dis J.	Greece	cohort study	≤15	13	TST	0.85	–	≥5	Low
						QFT-IT	0.85^a^	–		
Cruz et al. (2011) [35]	Pediatrics.	USA	cohort study	≤18	13	TST	0.77	–	≥5	Low
						T-SPOT.TB	0.92	–		
Chiappini et al. (2012) [36]	PLoS One.	Italy	cohort study	≤18	5 29	TST	0.80^a^	0.97^a^	≥5	Low
						QFT-IT	0^a^	1^a^		
Lodha et al. (2013) [37]	Int J Tuberc Lung Dis.	India	RCT	6 m - 15	128	TST	0.90^a^	–	≥10	High
						QFT-IT	0.83^a^	–		
Blandinières et al. (2013) [38]	J Infect.	France	case–control study	≤15	24	TST	0.78^a^	–	≥10	Low
						QFT-IT	0.70^a^	–		
Jenum et al. (2014) [39]	Pediatr Infect Dis J.	India	cohort study	9 m -28 m	4 692	TST	0.75^a^	0.91^a^	≥10	High
						QFT-IT	0.75^a^	0.95^a^		
Chiappini et al. (2014) [40]	Pediatr Infect Dis J.	Italy	cohort study	<18	28 210	TST	0.96^a^	–	–	Low
						QFT-IT	0.89^a^	0.96^a^		
						T-SPOT.TB	0.78^a^	0.99^a^		
Petrone et al. (2015) [41]	Biomed Res Int.	Uganda	cohort study	1 m - 16	7	TST	0.50^a^	–	≥10	High
						QFT-IT	0.60^a^	–		

^a^ Sensitivity and specificity were not directly reported and were calculated from available data in the study

*RCT* randomized controlled trial

### Quality assessment

**Fig. 2:**
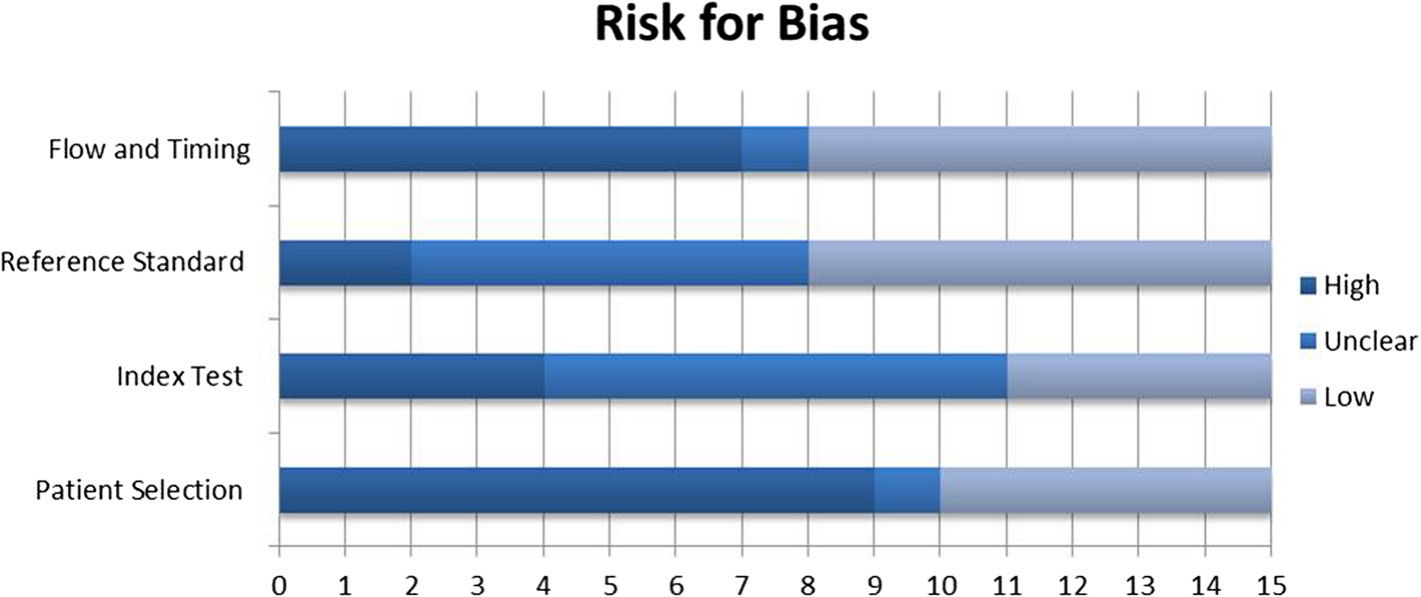
Results of quality assessment according to the QUADAS-2 tool

Results of the quality assessment are summarized in Table 2 and Fig. 2. Before disagreements were resolved, reviewers’ consensus on risk for bias and concerns regarding applicability were 91.7 and 97.7 %, respectively. No study was considered at low risk for bias in all the domains while all studies scored low in terms of concerns regarding applicability in all domains. The study of Sun Lin et al. [33] and that of Cruz et al. [35] were considered to be the most at risk of bias; judged at high risk in each domain with the exception of the Index Test domain (Sun Lin et al.) and Reference Standard domain (Cruz et al.). The studies of Detjen et al. [27], Kampmann et al. [29], Hansted et al. [31], and Chiappini et al. [36] were considered to be less at risk for bias; judged at low risk in each domain with the exception of the Patient Selection domain (Detjen et al. [27], Kampmann et al. [29]) and Index Test domain (Hansted et al. [31] and Chiappini et al. [23, 36]).

**Table 2:** Results of the quality assessment according to the QUADAS-2 tool

	Risk for bias				Applicability concerns		
Study	Patient selection	Index test	Reference standard	Flow and timing	Patient selection	Index test	Reference standard
Detjen et al. (2007) [27]	H	L	L	L	L	L	L
Domínguez et al. (2008) [28]	U	H	L	U	L	L	L
Kampmann et al. (2009) [29]	H	L	L	L	L	L	L
Lighter et al. (2009) [30]	H	U	U	L	L	L	L
Hansted et al. (2009) [31]	L	H	L	L	L	L	L
Bamford et al. (2010) [32]	H	L	L	H	L	L	L
Sun Lin et al. (2010) [33]	H	L	H	H	L	L	L
Tsolia et al. (2010) [34]	L	U	H	H	L	L	L
Cruz et al. (2011) [35]	H	H	L	H	L	L	L
Chiappini et al. (2012) [36]	L	H	L	L	L	L	L
Lodha et al. (2013) [37]	H	U	U	L	L	L	L
Blandinières et al. (2013) [38]	H	U	U	L	L	L	L
Jenum et al. (2014) [39]	L	U	U	H	L	L	L
Chiappini et al. (2014) [40]	L	U	U	H	L	L	L
Petrone et al. (2015) [41]	H	U	U	H	L	L	L

*H* high risk for bias, *U* unclear risk for bias, *L* low risk for bias

In the Patient Selection domain (domain 1), five studies scored low risk for bias, one scored unclear risk (recruitment protocol not clearly stated), and nine scored high risk (sample of patients enrolled in a non-consecutive, non-random way or inappropriate exclusions not avoided). In the Index Test domain (domain 2), four studies had low risk for bias, in seven cases it was unclear whether the index test results were interpreted with or without knowledge of the results of the reference standard, and four scored high risk (the index test results interpreted with knowledge of the results of the reference standard). In the Reference Standard domain (domain 3), seven studies showed low risk for bias, six had unclear risk, and two were judged at high risk for bias. Indeed, in six studies, it was unclear if results of the reference standard were interpreted without knowledge of the index test, and in two cases reviewers judged that results of the reference standard were interpreted with knowledge of the index test. In the Flow and Timing domain (domain 4), seven studies scored low risk for bias, while seven were judged at high risk for bias, because not all patients recruited into the study were included in the analysis, and one scored unclear.

### Diagnostic performance

TST (cut-off stated in the study), QFT-IT and T-SPOT TP, TN, FP, and FN for each study are reported in Table 3.

**Table 3:** Results of TST, QFT-IT and T-SPOT.TB

Author, Year	TST (cut-off stated in each study)				QFT-IT					T-SPOT.TB				
	TP	TN	FP	FN	TP	TN	FP	FN	IND	TP	TN	FP	FN	IND
Detjen et al. (2007) [27]	28	22	0	0	26	21	0	2	1	26	21	0	2	1
Domínguez et al. (2008) [28]	9	–	–	0	6	–	–	3	0	6	–	–	1	2
Kampmann et al. (2009) [29]	21	–	–	3	20	–	–	3	2	14	–	–	9	1
Lighter et al. (2009) [30]	6	18	3	1	6	21	0	1	0	/	/	/	/	/
Hansted et al. (2009) [31]	23	18	34	0	/	/	/	/	/	23	47	5	0	0
Bamford et al. (2010) [32]	37	–	–	8	36	–	–	6	4	18	–	–	8	1
Sun Lin et al. (2010) [33]	11	36	15	7	/	/	/	/	/	15	48	3	3	0
Tsolia et al. (2010) [34]	11	–	–	2	11	–	–	0	2	/	/	/	/	/
Cruz et al. (2011) [35]	10	–	–	3	/	/	/	/	/	12	–	–	1	0
Chiappini et al. (2012) [36]	4	28	1	1	0	29	0	5	0	/	/	/	/	/
Lodha et al. (2013) [37]	115	–	–	13	102	–	–	21	5	/	/	/	/	/
Blandinières et al. (2013) [38]	18	–	–	5	16	–	–	7	0	/	/	/	/	/
Jenum et al. (2014) [39]	3	624	65	1	3	625	33	1	20	/	/	/	/	/
Chiappini et al. (2014) [40]	27	–	–	1	25	195	9	3	0	21	187	2	6	17
Petrone et al. (2015) [41]	3	–	–	3	3	–	–	2	0	/	/	/	/	/

*TN* true negatives, *TP* true positives, *FN* false negatives, *FP* false positives, *IND* indeterminate

It was only possible to define TP, TN, FP and FN according to the three TST cut off of AAP for two studies (Detjen et al. [27], Chiappini et al. [23, 36]) (Table 4).

**Table 4:** Results of TST (according to the three TST cut off of AAP)

Author, Year	AAP cut off > 5				AAP cut off > 10				AAP cut off > 15			
	TP	TN	FP	FN	TP	TN	FP	FN	TP	TN	FP	FN
Detjen et al. (2007) [27]	28	22	0	0	28	22	0	0	17	22	0	11
Chiappini et al. (2012) [36]	4	28	1	1	4	29	0	1	3	29	0	2

*TN* true negatives, *TP* true positives, *FN* false negatives, *FP* false positives

### Pooled sensitivity and specificity

#### Accuracy of TST

**Fig. 3:**
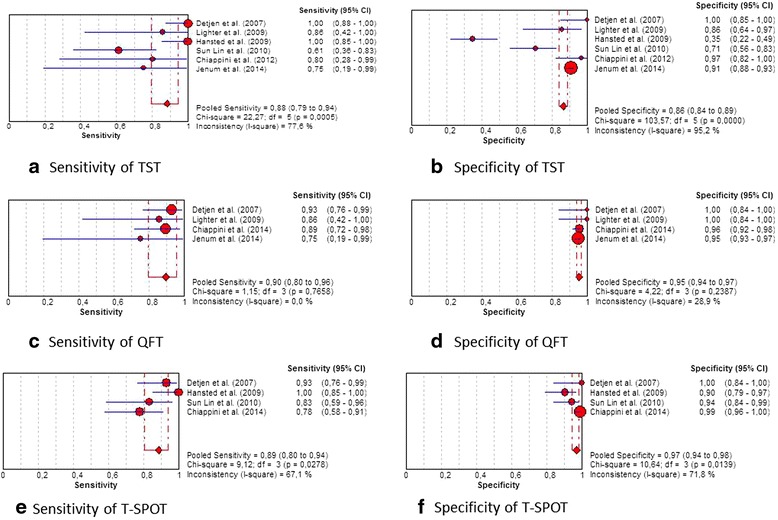
Plot of individual studies and pooled estimates of sensitivity and specificity. **a** sensitivity of TST, **b** specificity of TST, **c** sensitivity of QFT, **d** specificity of QFT, **e** sensitivity of T-SPOT, **f** specificity of T-SPOT

Among six studies (Detjen et al. [27], Lighter et al. [30], Hansted et al. [31], Sun Lin et al. [33], Chiappini et al. [23, 36], Jenum et al. [39]), the overall sensitivity of the TST (with respect to cut-off stated in each study) was 88.2 % (95 % CI 79.4–94.2 %). Of note, the degree of heterogeneity of the studies was high (I^2^ = 77.6 %) (Fig. 3a). The pooled specificity was 86.3 % (95 % CI 83.9–88.6 %). Even here the degree of heterogeneity between the studies was high (I^2^ = 95.2 %) (Fig. 3b). Pooled LR+ and LR- were 5.3 and 0.2, respectively and the AUC was 0.925. Among the two studies (Detjen et al. [27], Chiappini et al. [23, 36]) where calculation was possible, the overall sensitivity of the TST, with respect to the AAP cut-off of >5 mm, was 97.0 % (95 % CI 84.2–99.9 %) with a heterogeneity of 74.7 %. The pooled specificity was 98.0 % (95 % CI 89.6–100 %) with a heterogeneity of 12.6 %. Using the AAP cut-off of >10 mm, the overall sensitivity of the TST was 97.0 % (95 % CI 84.2–99.9 %) with a heterogeneity of 74.7 %; the pooled specificity was 100 % (95 % CI 93.0–100.0 %) with a heterogeneity of 0 %. According to the AAP cut off > 15 mm, the overall sensitivity of the TST was 60.6 % (95 % CI 42.1–77.1 %) with a heterogeneity of 0 %; the pooled specificity was 100 % (95 % CI 93.0–100.0 %) with a heterogeneity of 0 %.

#### Accuracy of the QFT-IT assay

Four studies (Detjen et al. [27], Lighter et al. [30], Chiappini et al. [40], Jenum et al. [39]) included the QFT- IT assay with a pooled sensitivity of 89.6 % (95 % CI 79.7–95.7 %) (Fig. 3c). Heterogeneity between the studies was absent (I^2^ = 0 %). The pooled specificity was 95.4 % (95 % CI 93.8–96.6 %) (Fig. 3d) with slow heterogeneity (I^2^ = 28.9 %). Pooled LR+ and LR- were 18.2 and 0.1, respectively and AUC was 0.988.

#### Accuracy of the T-spot assay

For determining sensitivity of the T-SPOT, four studies (Detjen et al. [27], Hansted et al. [31], Sun Lin et al. [33], Chiappini et al. [40]) were included in the analysis. It resulted in a pooled sensitivity of 88.5 % (95 % CI 80.4–94.1 %) (Fig. 3e). The degree of heterogeneity was 67.1 %. The pooled specificity was of 96.8 % (95 % CI 94.2–98.5 %) with a heterogeneity of 71.8 % (Fig. 3f). Pooled LR+ and LR- were 20.4 and 0.2 respectively and the AUC was 0.978.

## Discussion

Our study demonstrates that all the three tests were highly accurate as shown by the AUC. According to the confidence intervals of pooled estimates, there are no significant differences in sensitivity among the three methodologies assessed: TST pooled sensitivity: 88.2 %, 95 % CI 79.4–94.2 %; QFT-IT pooled sensitivity: 89.6 %, 95 % CI 79.7–95.7 %; and T-SPOT pooled sensitivity: 88.5 %, 95 % CI 80.4–94.1 %. However, with respect to specificity, both QFT-IT (pooled specificity: 95.4 %, 95 % CI 93.8–96.6 %) and T-SPOT (pooled specificity: 96.8 %, 95 % CI 94.2–98.5 %) performed significantly better than TST (pooled specificity: 86.3 %, 95 % CI 83.9–88.6 %). Subsequently, our findings highlight that IGRAs have a higher specificity than TST for detecting active TB cases in immunocompetent children.

For sensitivity, our results are consistent with the recent findings of Sollai et al. [24] (TST pooled sensitivity: 79 %, 95 % CI 75–83 %; QFT-IT pooled sensitivity: 81 %, 95 % CI 76–85 %; T-SPOT pooled sensitivity: 80 %, 95 % CI 74–84 %). Moreover, with respect to the previously published meta-analysis, we have provided additional evidence of a higher specificity of QFT-IT and T-SPOT in bacteriologically confirmed active TB in immunocompetent children.

Since the sensitivity is equal, this improved specificity of QFT-IT and T-SPOT ensures that healthy children are not wrongly diagnosed as an active TB patient and incorrectly treated as such, exposing them to two or three drugs for at least six months. This improved specificity also reduces the negative emotional impact of a false positive result on the families of children.

The diagnosis of active TB in children is especially problematic as symptoms can be confused with those of common childhood diseases and sputum samples are harder to obtain. For these reasons, and because of the higher specificity we have shown, the IGRAs could be used as complementary tests to support the clinical diagnosis of active TB, in particular in the absence of bacteriological confirmation. However, it should always be considered that a negative IGRA, as well as a negative TST result, does not exclude active TB. This may be appreciated by looking at LR+ and LR- which provide an idea of the utility of the test. All the three tests we assessed have similar LR- but different LR+ (5.3 for TST, 18.2 for QFT, and 20.4 for T-SPOT). This means that if the ratio of the odds of having a negative test result in a TB patient to the odds of the same result in a healthy one is similar for the three tests, the ratio of the odds of having a positive test result in a diseased patient to the odds of the same result in a healthy child is much higher using QFT and T-SPOT instead of TST. This makes these tests useful in clinical practice as they allow clinicians to make a diagnosis of active TB [42].

The improved specificity in healthy children confirms previous evidence [12, 43, 44], encouraging the primary use of QFT-IT or T-SPOT for case finding among healthy children and young patients [45]. These children may also fail to present for TST reading as previously suggested by Lewinsohn et al. [8]. From a Public Health perspective, our results provide an opportunity to consider the use of these tests in screening too. In fact, even though all the tests we have assessed showed similar sensitivities, IGRAs do not require, unlike TST, a second visit to assess results, which may be problematic for large and specific populations [46]. Furthermore, IGRAs have been suggested to be more accurate than TST in immunocompetent people [47] and allow distinguishing individuals who have been previously vaccinated, which could represent an advantage for screening. In fact, IGRAs have already been used to screen children during the investigation of potentially exposed newborns in a Teaching Hospital [48] and the use of IGRAs in “one step” approach has been also proposed in other contexts [49, 50].

### Limitations

Our study has a number of limitations. First, of all the studies fulfilling our inclusion criteria considered small populations. There is a small number of published studies focused on children, especially those aged <5 years. In fact, caution should be exercised when considering the preferential use of IGRAs in immunocompetent children aged <5 years; a warning to this effect was added to the national guidelines in the United States in a recent update [51]. Another limitation is the heterogeneity of studies, particularly concerning different and specific age groups. We did not perform a sub-analysis according to the size of TB burden (low versus high) because of the small number of studies we were able to include. For the same reason, a funnel plot was not used to investigate potential publication bias. The indeterminate rate results (inadequate interferon-γ response to positive control (PHA/mitogen) due to anergy, excessive interferon-γ in the negative control or, only for T-SPOT, insufficient cells, < 250,000 cells/100 μl) among children, which is considered an important impediment to the use of IGRAs in clinical practice for children [51], was not available in all the included studies. Further research should focus on evaluating the additional value of safety, social and ethical implications, organizational impact, and cost-effectiveness of IGRAs on the basis of a Health Technology Assessment approach.

## Conclusions

QFT-IT and T-SPOT have a higher specificity than TST for detecting active TB cases in immunocompetent children, providing evidence for choices available to clinicians. These tests may be used as complementary tests to support the clinical diagnosis of active TB and may be also considered as part of public health responses.

## Abbreviations

AAP: American Academy of Pediatrics
CI: confidence interval
ELISA: enzyme-linked immunosorbent assay
ELISPOT: enzyme-linked immunosorbent spot
FN: false negatives
FP: false positives
HIV: human immunodeficiency virus
IFN: interferon
IGRA: interferon-γ release assay
IND: indeterminate
LR+: likelihood positive ratio
LR−: likelihood negative ratio
LTBI: latent tuberculosis infection
*M. tuberculosis*: *Mycobacterium tuberculosis*
QFT-G: QuantiFERON-TB Gold
QFT-IT: QuantiFERON-TB Gold In-Tube
QUADAS: Quality Assessment of Diagnostic Accuracy Studies
TB: tuberculosis
TN: true negatives
TP: true positives
TST: Tuberculin Skin Test
UK: United Kingdom
